# AAV‐Mediated Gene Therapy Restores Hearing in Patients with DFNB9 Deafness

**DOI:** 10.1002/advs.202306788

**Published:** 2024-01-08

**Authors:** Jieyu Qi, Fangzhi Tan, Liyan Zhang, Ling Lu, Shanzhong Zhang, Yabo Zhai, Yicheng Lu, Xiaoyun Qian, WenXiu Dong, Yinyi Zhou, Ziyu Zhang, Xuehan Yang, Lulu Jiang, Chaorong Yu, Jiancheng Liu, Tian Chen, Lianqiu Wu, Chang Tan, Sijie Sun, Huaien Song, Yilai Shu, Lei Xu, Xia Gao, Huawei Li, Renjie Chai

**Affiliations:** ^1^ State Key Laboratory of Digital Medical Engineering Department of Otolaryngology Head and Neck Surgery Zhongda Hospital School of Life Sciences and Technology School of Medicine Advanced Institute for Life and Health Jiangsu Province High‐Tech Key Laboratory for Bio‐Medical Research Southeast University Nanjing 210096 China; ^2^ Co‐Innovation Center of Neuroregeneration Nantong University Nantong 226001 China; ^3^ Department of Neurology, Aerospace Center Hospital, School of Life Science Beijing Institute of Technology Beijing 100081 China; ^4^ Department of Otolaryngology‐Head and Neck Surgery the Affiliated Drum Tower Hospital of Nanjing University Medical School Jiangsu Provincial Key Medical Discipline (Laboratory) Nanjing 210008 China; ^5^ Otovia Therapeutics Inc Suzhou 215101 China; ^6^ School of Medicine Southeast University Nanjing 210009 China; ^7^ Fosun Health Capital Shanghai 200233 China; ^8^ ENT Institute and Department of Otorhinolaryngology Eye & ENT Hospital State Key Laboratory of Medical Neurobiology and MOE Frontiers Center for Brain Science Fudan University Shanghai 200031 China; ^9^ Institute of Biomedical Science Fudan University Shanghai 200032 China; ^10^ NHC Key Laboratory of Hearing Medicine Fudan University Shanghai 200032 China; ^11^ Department of Otolaryngology‐Head and Neck Surgery Shandong Provincial ENT Hospital Shandong University Jinan Shandong 250022 China; ^12^ The Institutes of Brain Science and the Collaborative Innovation Center for Brain Science Fudan University Shanghai 200032 China; ^13^ Department of Otolaryngology Head and Neck Surgery Sichuan Provincial People's Hospital University of Electronic Science and Technology of China Chengdu 610072 China; ^14^ Southeast University Shenzhen Research Institute Shenzhen 518063 China

**Keywords:** AAV, biosafety, clinical trial, hearing recovery, OTOF gene therapy

## Abstract

Mutations in OTOFERLIN (*OTOF*) lead to the autosomal recessive deafness 9 (DFNB9). The efficacy of adeno‐associated virus (AAV)‐mediated *OTOF* gene replacement therapy is extensively validated in *Otof*‐deficient mice. However, the clinical safety and efficacy of AAV‐*OTOF* is not reported. Here, AAV‐*OTOF* is generated using good manufacturing practice and validated its efficacy and safety in mouse and non‐human primates in order to determine the optimal injection dose, volume, and administration route for clinical trials. Subsequently, AAV‐*OTOF* is delivered into one cochlea of a 5‐year‐old deaf patient and into the bilateral cochleae of an 8‐year‐old deaf patient with *OTOF* mutations. Obvious hearing improvement is detected by the auditory brainstem response (ABR) and the pure‐tone audiometry (PTA) in these two patients. Hearing in the injected ear of the 5‐year‐old patient can be restored to the normal range at 1 month after AAV‐*OTOF* injection, while the 8‐year‐old patient can hear the conversational sounds. Most importantly, the 5‐year‐old patient can hear and recognize speech only through the AAV‐*OTOF*‐injected ear. This study is the first to demonstrate the safety and efficacy of AAV‐*OTOF* in patients, expands and optimizes current *OTOF*‐related gene therapy and provides valuable information for further application of gene therapies for deafness.

## Graphical abstract

This study evaluates the efficacy and safety of an adeno‐associated virus (AAV) based gene therapy AAV‐*OTOF* in patients with DFNB9 deafness. AAV‐*OTOF* injection can restore the hearing function of the previous deaf ear to a normal level with safety. This proof of concept study provides clinical gene therapy data for DFNB9 deafness and also supplies support for other inner ear gene therapies.

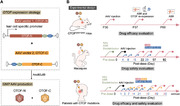

## 1 Introduction

Hearing impairment shows the highest incidence among sensory defects in human populations, and genetic mutations are important causes of deafness. Although more than 150 gene mutations have been identified to cause deafness, no medical treatment is available to treat the deafness caused by any of the mutations. Auditory neuropathy (AN) is a kind of hearing loss with autosomal recessive inheritance. Zhang et al. found that *OTOF* mutations are a major reason for infantile auditory neuropathy DFNB9.^[^ ^1^ ^]^ In mature cochlear tissues, OTOF is expressed in the pre‐synaptic ribbons of inner hair cells (IHCs).^[^ ^2^ ^]^ OTOF interacts with calcium, SNAP25, and Synataxin‐1 to mediate the exocytotic release of the excitatory neurotransmitter glutamate.^[^ ^2^, ^3^ ^]^ *OTOF* mutations lead to reduced transmission efficiency between IHCs and spiral ganglion neurons, as well as the loss of synchronous firing of spiral ganglion neurons, thus resulting in hearing loss and speech discrimination disorders.^[^ ^4^ ^]^ Homozygous and compound heterozygous *OTOF* mutations mostly cause severe to profound prelingual deafness and temperature‐sensitive deafness.^[^ ^5^, ^6^, ^7^ ^]^

Currently, cochlear implant is the only effective treatment that is recommended to DFNB9 patients with severe to profound deafness.^[^ ^8^ ^]^ However, the restored hearing is poorer than the natural hearing in many aspects due to the limitations of this technology, including poor tone recognition, difficulty in processing intonation, and inability to appreciate music.^[^ ^9^, ^10^ ^]^ Thus, other treatments are still needed to restore natural hearing.

Gene therapy is currently considered the most promising strategy to cure genetic diseases such as inherited hearing loss. More than 40 studies have reported successful hearing restoration via gene therapy in animal models, with each focused on a deaf mutation among 20s genes.^[^ ^11^ ^]^ Gene therapy treats genetic diseases by manipulating target genes via different methods including gene replacement, gene suppression, and gene editing strategies. Gene replacement, a dominant strategy in clinical studies, provides sufficiently functional protein by delivering exogenous genes, and this method is suitable for the treatment of recessive genetic diseases and dominant genetic diseases that have insufficient single allele dosage, such as *OTOF* mutation‐induced autosomal recessive DFNB9.

The gene replacement therapy for treating DFNB9 has passed the proof‐of‐concept stage in mouse models.^[^ ^12^, ^13^, ^14^, ^15^, ^16^, ^17^, ^18^, ^19^, ^20^, ^21^, ^22^, ^23^, ^24^, ^25^, ^26^ ^]^ It is a good timing now to initiate the clinical trial for bench‐to‐bedside translation. AAV mediated gene therapy has been the most promising strategy for monogenic inherited disorders, and several AAV gene therapy products have been approved by the US FDA, but there is not yet any AAV product for treating deafness. With the screening and identification of AAV serotypes targeting IHCs, such as Anc80L65, PhP.B, AAV1, etc.,^[^ ^27^, ^28^, ^29^, ^30^ ^]^ AAV‐based gene therapy has become a promising potential treatment to reverse *OTOF* mutation‐induced deafness.

The human full‐length *OTOF* gene is ≈6 kb, exceeding the packaging capacity of AAV (≈4.7 kb),^[^ ^31^ ^]^ and dual‐AAV delivery strategies based on DNA or protein recombination have helped to solve the problem of delivering *OTOF* into mice.^[^ ^22^, ^23^, ^32^ ^]^ Therapeutic experiments in *Otof*‐deficient mice using dual AAVs to re‐express full‐length OTOF showed that hearing loss could be partially or completely restored in both neonatal and adult mice,^[^ ^22^, ^23^, ^32^ ^]^ indicating the clinical application potential of dual‐AAV‐*OTOF*. However, relevant preclinical studies on the safety and optimal dosage in large animal models have not been reported, nor have there been any efficacy and safety evaluations in patients with *OTOF* mutations.

In this study, we used an AAV serotype with a high transduction rate for IHCs, along with a hair cell‐specific promoter, to generate dual AAV‐*OTOF* vectors (AAV‐*OTOF*‐N and AAV‐*OTOF*‐C) conforming to the good manufacturing practice of medical products (GMP), which together produce full‐length OTOF in hair cells. We first validated the efficacy of AAV‐*OTOF* at restoring damaged hearing in homozygous OTOF^Q939*/Q939*^ mice and verified the systemic safety of AAV‐*OTOF* and inner ear administration in cynomolgus macaques. AAV‐*OTOF* could restore hearing loss in deaf OTOF^Q939*/Q939*^ mice and did not cause hearing changes or systemic toxicity related to the drug or administration approach in either wildtype mice or cynomolgus macaques. Based on the animal studies, we determined the clinical trial scheme and performed an exploratory clinical trial in two children (a 5‐year‐old and an 8‐year‐old) carrying *OTOF* mutations to assess the efficacy and safety of AAV‐*OTOF*. AAV‐*OTOF* was injected into the cochleae through the round window of the patients, and we analyzed the auditory function of the patients and tested neutralizing antibodies against AAV‐*OTOF*, measured the blood AAV biodistribution, and performed blood cell biochemistry, blood biochemistry, blood coagulation, and urinalysis. The enrolled patients showed effective improvement on hearing from 2 weeks after surgery, with the best hearing thresholds recovering from >100 dB to the normal range (30 dB) at 1 month in the objective tone‐burst ABR results. More importantly, the AAV‐*OTOF* transduced ear of the 5‐year‐old child gained comparable hearing to her contralateral ear, which had a cochlear implant. In addition, 3‐month follow‐up did not show any evidence of adverse effect related to AAV‐*OTOF*. These results demonstrate a promising future for AAV mediated *OTOF* delivery as a treatment for DFNB9.

### 1.1 Study Design

GMP‐grade AAV‐*OTOF* was manufactured and the titers of AAV‐*OTOF*‐N and AAV‐*OTOF*‐C were both 2.8 × 10^13^ vg mL^−1^ (vg: viral genomes). AAV‐*OTOF* was subjected a number of checks, including sterility and the presence of bacterial endotoxin, mycoplasma, or chemical residues from the production and purification process such as iodixanol, host residues, etc. All inspection indicators for AAV‐*OTOF* met the production release criteria.

**Figure 1:**
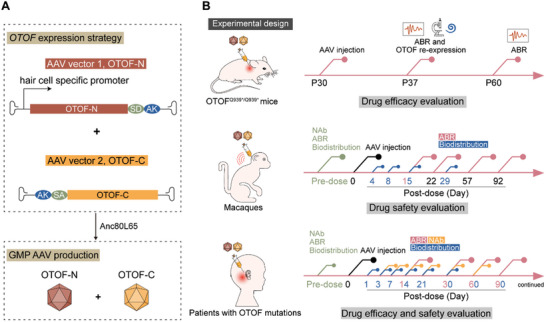
Experimental design. A) Human *OTOF* expression method. Full‐length *OTOF* was packaged into dual AAV vectors and acquired by DNA recombination via the AK sequence and RNA trans‐splicing via the splicing donor (SD) and splicing acceptor (SA) after delivery. *OTOF* expression was driven by the mouse hair cell‐specific promoter. The therapeutic AAVs were manufactured in accordance with the GMP level. B) The experimental and therapeutic design of AAV‐*OTOF* therapy in mice, cynomolgus macaques, and patients with *OTOF* mutations.

In biological studies, we first validated the efficacy of the GMP‐grade AAV‐*OTOF* containing AAV‐*OTOF*‐N and AAV‐*OTOF*‐C at restoring hearing in deaf OTOF^Q939*/Q939*^ mice. We then performed hearing and systemic safety verification of the drug in both wild‐type mice and cynomolgus macaques using the same administration approach as in the clinical trials. After comprehensively evaluating the drug's efficacy and safety, we conducted a single center, open‐label, individual‐patient, non‐randomized controlled interventional clinical study by performing a single round window injection of AAV‐*OTOF* in two pediatric patients with profound deafness caused by *OTOF* mutations (**Figure** **1**). Detailed diagnoses and hearing assessments were performed before treatment, and informed consent was obtained from the patient's guardians prior to enrollment. This study was approved by the Clinical Research Ethics Committee of Shandong Second Provincial General Hospital (ethical approval number: 2023‐010‐03) and registered on ClinicalTrials.gov, No. NCT05901480.

### 1.2 Cases Report

By using whole exome sequencing, the compound heterozygous mutation c.4275G>A (p.W1425X) and c.2377_2401del (p.E793Sfs*16) in *OTOF* was identified in patient 1 (5 years old). Patient 2 (8 years old) carried the compound heterozygous mutation c.1917G>A (p.W639X) and c.1066T>G (p.W356G) in *OTOF*. The 5‐year‐old patient wore hearing aids and received speech training for 6 months at 3 years of age, with only poor hearing improvement. In November 2021, patient 1 underwent cochlear implantation (CI) in the right ear. With ethics approval and parental consent, the pediatric patients participated in an individual‐patient expanded‐access clinical trial at Shandong Second Provincial General Hospital to receive the investigational AAV‐*OTOF* gene replacement therapy. In August 2023, AAV‐*OTOF* was injected into the left cochlea of the 5‐year‐old patient. In September 2023, AAV‐*OTOF* was injected into the bilateral cochleae of the 8‐year‐old patient.

## 2 Results

### 2.1 AAV‐OTOF Effectively Restored Hearing to Almost Wild‐Type Levels in Adult OTOFQ939*/Q939* Mutant Mice

**Figure 2:**
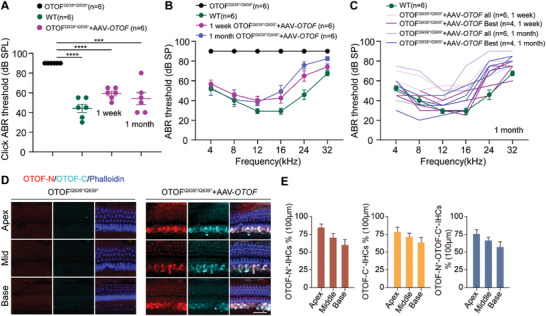
AAV‐*OTOF* delivery efficiently restored hearing in adult OTOF^Q939*/Q939*^ mice. A) The click ABR results in OTOF^Q939*/Q939*^ mice, wild type (WT) mice, and the P30 OTOF^Q939*/Q939*^ mice injected with AAV‐*OTOF* after 1 week and 1 month, respectively. B) The ABR results from OTOF^Q939*/Q939*^ mice, WT mice, and the P30 OTOF^Q939*/Q939*^ mice injected with AAV‐*OTOF* after 1 week and 1 month, respectively. C) The ABR results of WT mice, all OTOF^Q939*/Q939*^ mice, and the best performing OTOF^Q939*/Q939*^ mice injected with AAV‐*OTOF* after 1 week and 1 month, respectively. D) The confocal images of *OTOF*‐N and *OTOF*‐C in adult OTOF^Q939*/Q939*^ mice injected with AAV‐*OTOF* after 7 days. *OTOF*‐N: red, *OTOF*‐C: cyan, phalloidin: blue. Scale bar: 50 µm. E) The percentage of OTOF‐positive IHCs in (D). All data are shown as the mean ± SEM. The *p*‐value was calculated by Student's *t*‐test. ***p < 0.001 and ****p < 0.0001.

We studied the efficacy of GMP‐grade AAV‐*OTOF* in adult OTOF^Q939*/Q939*^ point mutant mice. The mixed AAV‐*OTOF* viruses (2.8 × 10^10^ vg per AAV) were injected into the cochleae of deaf postnatal day (P) 30 OTOF^Q939*/Q939*^ mice, and ABR testing at 1 week after injection showed significant improvement (**Figure** **2**A–C). The hearing of most of the treated *OTOF* mutant mice in the middle‐to‐low frequencies was comparable with that of wild‐type mice (Figure 2C). Longer‐term observation in AAV‐*OTOF*‐injected mice showed a relatively stable hearing recovery (Figure 2A–C). In addition, immunofluorescence staining was performed to detect the recombinant OTOF expression in OTOF^Q939*/Q939*^ mice by using antibodies against the OTOF N‐ and C‐terminus, respectively. The results showed effective full‐length OTOF expression in IHCs (Figure 2D) with a recombination efficiency up to 75% (Figure 2E). These data indicated that AAV‐*OTOF* can effectively restore hearing in adult OTOF^Q939*/Q939*^ mice, suggesting a wide therapeutic window and promising application potential in the clinic.

### 2.2 Biological Safety Evaluation of AAV‐*OTOF* in Mice and Cynomolgus Macaques

To evaluate the AAV dosage in the clinical trial, we further explored the potential toxic reactions and AAV biodistribution after AAV‐*OTOF* injection in mice and cynomolgus macaques. We injected AAV‐*OTOF* into the inner ears of 4‐week‐old wild‐type mice and evaluated their hearing, body weight, and neurological and pathological changes at 3 months after injection. Mice of both sexes were divided into four groups, including blank wild‐type, menstruum‐injected, low dose (7.0 × 10^9^ vg per AAV), and high dose (2.8 × 10^10^ vg per AAV). ABR testing showed that the therapeutic dose used in the treatment of OTOF^Q939*/Q939*^ mutant mice (Figure 2) did not affect hearing in wild‐type mice (Figure S1A,B, Supporting Information). In addition, weight gain in the mice was also unaffected (Figure S1C, Supporting Information), and behavioral experiments confirmed that AAV‐*OTOF* injection did not affect locomotor or memory abilities (Figure S1D, Supporting Information). Unexpectedly, we found that AAVs could enter the circulatory system and thus distributed to other organs, especially the liver, in a dose‐dependent manner (Table S1, Supporting Information). But blood cell counts and blood biochemistry showed no clinically significant changes, indicating that AAVs in blood did not exhibit detectable toxicity.

**Figure 3:**
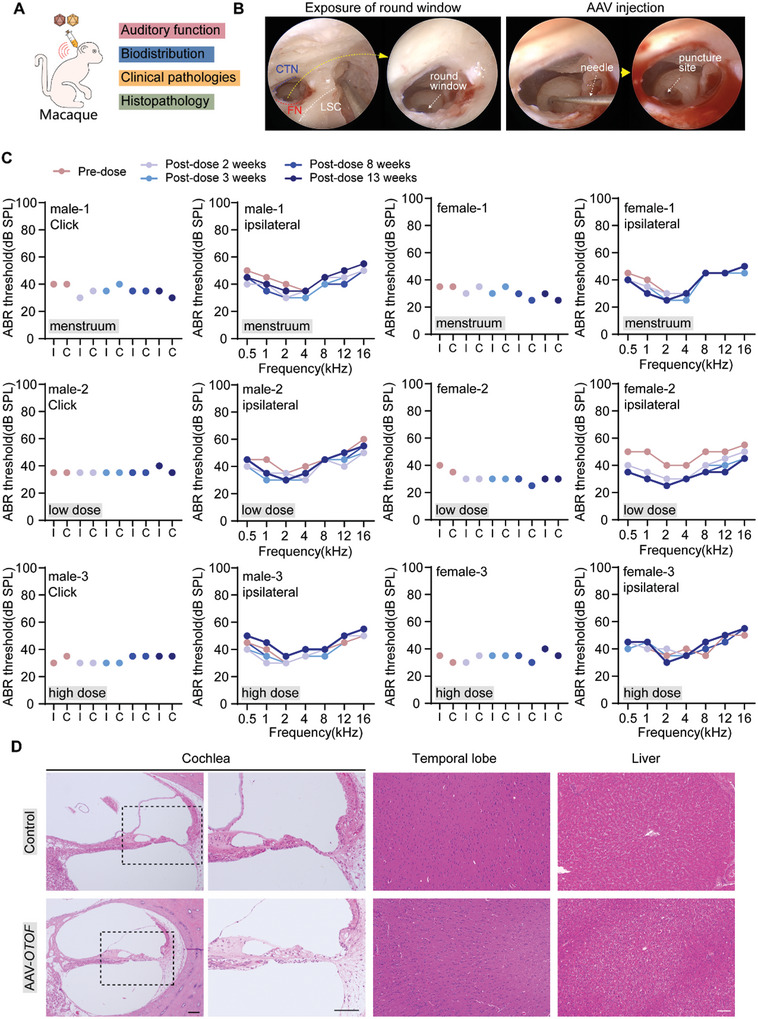
AAV‐*OTOF* was audiologically and systemically safe in cynomolgus macaques. A) The experimental procedure in cynomolgus macaques. B) The images of round window membrane exposure and AAV injection in cynomolgus macaques. C) The ABR results of click and tone‐burst in cynomolgus macaques injected with menstruum, low dose AAV‐*OTOF*, and high dose AAV‐*OTOF*, respectively. I: ipsilateral ear, C: contralateral ear. D) The HE staining of tissues from control and AAV‐*OTOF* injected cynomolgus macaques. Scale bar: 100 µm.

To further evaluate the biological safety of GMP‐grade AAV‐*OTOF*, we explored potential physiological and pathological changes induced by injection of different doses in cynomolgus macaques. Detailed body morphology observations, body weight, temperature, blood cell counts, coagulation, blood biochemistry, neutralizing antibodies against AAV vector, and auditory function were all examined prior to dosing to qualified animals. Cynomolgus macaques aged 3–5 years and weighting 2–5 kg were enrolled. There were six monkeys with equal numbers of males and females, and these were divided into the menstruum group, low dose AAV‐*OTOF* group (1/4 dose), and high dose group (1× dose, 4.2 × 10^11^vg per AAV), with one male and one female per group. Observations, body weight, temperature, hearing tests, biodistribution of viral vectors, and neutralizing antibodies against AAV were assessed at various time points after dosing (**Figure** **3**A). AAV dosing was performed by round window membrane injection through the trans‐mastoid facial recess (Figure 3B; Movie S1, Supporting Information). *OTOF*‐N and *OTOF*‐C DNA could be detected in a dose‐dependent manner in blood distribution on the first day after injection (Table S2, Supporting Information). At 4–8 days after injection, AAV vector amounts in whole blood were below the detection limit (50 copies µg^−1^) (Table S2, Supporting Information). No deaths or moribund cases occurred in the study. No abnormalities were observed before or after dosing in terms of the body surface, visible mucous membranes, neurological or behavioral activities, the respiratory system, the digestive system, or the urinary or reproductive tract. Body weight and weight gain showed no abnormal changes. Most importantly, no abnormal changes in hearing function related to the surgery or AAV‐*OTOF* were observed. No significant differences in ABR thresholds were seen in each of the three groups between pre‐ and post‐surgery tests (Figure 3C), and the hearing function of the contralateral ear was also unchanged (Figure S2, Supporting Information). Together these findings indicated that we have established a safe inner ear delivery method that can potentially be used in the clinic.

Because AAV‐*OTOF* can enter the circulatory system, the tissue tolerance for AAV‐*OTOF* needs to be fully evaluated before clinical application. Thus, we performed hematoxylin‐eosin (HE) staining on tissue slices from different organs of cynomolgus macaques to assess systemic toxicity and inflammation. Histopathological analysis showed no morphological or pathological changes, inflammation, or fibrosis in animals injected with AAV‐*OTOF* (Figure 3D; Figure S3, Supporting Information). In summary, a single intracochlear injection of AAV‐*OTOF* did not cause significant toxic reactions in cynomolgus macaques. No adverse effects of surgery or drugs on hearing or systemic health were observed. The maximum dose used in the cynomolgus macaques was 8.4 × 10^11^vg per ear. This dosage regimen was used to design the dosing principles for clinical trials.

### 2.3 Almost Normal Hearing was Acquired in a 5‐Year‐Old Patient after AAV‐*OTOF* Administration in One Cochlea

**Figure 4:**
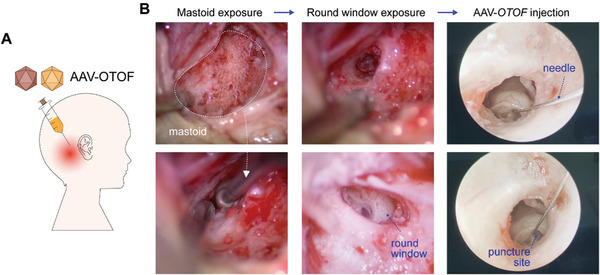
AAV‐*OTOF* was delivered into the cochlea through the trans‐mastoid facial recess. A) The experimental procedure in the patient. B) The images show the exposure of the round window membrane and AAV injection in the patient. The exposure of the mastoid and round window was performed under a surgical microscope, and injection of AAV‐*OTOF* was administrated via an oto‐endoscope.

Based on mouse and cynomolgus macaque data, we next determined the efficacy and safety of AAV‐*OTOF* in two patients of 5 and 8 years of age with auditory neuropathy due to *OTOF* mutations. Under general anesthesia, the round window was exposed via the trans‐mastoid facial recess and therapeutic AAV was delivered (**Figure** **4**; Movie S2, Supporting Information). The surgical procedure was consistent with that in cynomolgus macaques and did not damage cochlear structures or affect hearing (Figure 3C,D).

**Figure 5:**
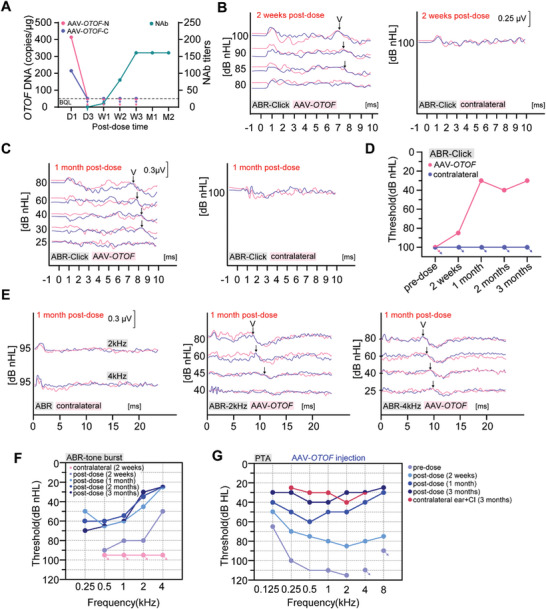
AAV‐*OTOF* rescued hearing in the 5‐year‐old patient with *OTOF* mutations. A) Genomic DNA copies of AAV‐*OTOF*‐N and AAV‐*OTOF*‐C and the NAbs levels from the patient at 1 day (D1), 3 days (D3), 1 week (W1), 2 weeks (W2), 3 weeks (W3), 1 month (M1) and 2 months (M2) after AAV injection, respectively. The arrows indicate that the DNA copies were below the quantification limit (BQL). B,C) Representative traces recorded for the click ABR from the patient at 2 weeks and 1 month after AAV‐*OTOF* injection. The recording was made twice at each sound intensity. Wave V is indicated by the arrows. D) The click ABR results of the patient. E) Representative traces recorded for tone‐burst ABR at 2 kHz and 4 kHz from the patient at 1 month after AAV‐*OTOF* injection. The recording was made twice at each sound intensity. Wave V is indicated by the arrows. F) The tone‐burst ABR results of the patient. G) The PTA results of the patient after AAV‐*OTOF* injection. And the PTA thresholds of the contralateral ears with the CI turning on were also shown. The arrows in (D, F, G) indicate that the patient had no responses to the maximum sound intensity at each frequency.

AAV‐*OTOF*‐N and AAV‐*OTOF*‐C DNAs were detected in whole blood on postoperative day 1 (414 copies/100 µL for *OTOF*‐N and 215 copies/100 µL for *OTOF*‐C) in the 5‐year‐old patient (**Figure** **5**A). This indicated that after injection into the inner ear AAV can rapidly spread to the blood vessels via lymphatic circulation. From day 3 onward, AAV was no longer detected in the blood, while the amount of neutralizing antibody (NAbs) in the serum began to increase (Figure 5A).

We next evaluated the auditory function of the patient at 2 weeks and 1, 2, and 3 months after surgery. Pre‐operatively, the 5‐year‐old patient passed the DPOAE evaluation for most frequencies (Figure S4A, Supporting Information). After surgery, the DPOAE declined at 2 weeks, with slightly gradual recovery with time (Figure S4A, Supporting Information). DPOAE is prone to false negatives, so in order to comprehensively evaluate hair cell function, cochlear microphonics (CM) was performed, and the results showed microphonic waves pre‐surgery and post‐ surgery at 2 weeks (Figure S5A, Supporting Information). We proposed that it still needs more time for the DPOAE recovery after surgery.

Next, we performed click ABR, tone‐burst ABR, and pure tone audiometry (PTA) to directly evaluate the recovery of auditory function. Wave V most closely represents the audiometric threshold. At 2 weeks after AAV‐*OTOF* injection, wave V of the click ABR could be elicited and recognized at 85 dB or greater intensities of sound stimulus (Figure 5B), indicating partial hearing recovery. The threshold of click ABR dramatically decreased to 30 dB at ≈1 month after surgery (Figure 5C,D), which was comparable to that of a normally hearing person. The ABR thresholds by 0.1 ms clicks best represents the hearing between 2 and 4 kHz due to the spectrum of the clicks and the speed of traveling wave difference across the cochlea, so click ABR does not reflect the hearing ability at the lower frequencies of human speech. Therefore, we performed tone‐burst ABR in the patient to determine the hearing recovery in a wider frequency range. Hearing recovery was in a time‐dependent manner. At 2 weeks after surgery, the ABR thresholds of the AAV‐*OTOF* injected ear were 90, 80, 80, and 50 dB at 0.5, 1, 2, and 4 kHz respectively, while the contralateral ear showed >95 dB at all frequencies; at 1 month after surgery, the ABR thresholds of the AAV‐*OTOF* injected ear were 50, 65, 60, 45, and 25 dB from 0.125 to 4 kHz, respectively (Figure 5E,F). Similar ABR thresholds were recorded at 2 months and 3 months after surgery (Figure 5F). Taken together, this patient acquired almost normal hearing after AAV‐*OTOF* injection.

Since ABR thresholds represent a gross estimation of hearing, we tested PTA to further evaluate the effect of the treatment on hearing function. Pre‐operatively, PTA thresholds at different frequencies were ≥100 dB except for 65 dB at 0.125 kHz in the left ear. At 2 weeks after surgery, PTA thresholds were partially decreased (threshold values: 50, 70, 75, 80, 85, 80, 75 dB at 0.125, 0.25, 0.5, 1, 2, 4, and 8 kHz, respectively) (Figure 5G). And, hearing thresholds continued to decrease significantly at 1 month (Figure 5G). We also tested the hearing ability with hearing aids. Before the surgery, the hearing improvement by the hearing aid was only 15–30 dB, not to a functional hearing to for effective communication (Figure 5G; Figure S6A, Supporting Information). One month after AAV‐*OTOF* injection, with the hearing aid, the hearing level of the injected ear was greatly improved compared to the preoperative hearing, and the hearing threshold improved from 70–95 dB to 30–35 dB, a level similar to that of the contralateral ear with CI (Figure S6A, Supporting Information). Excitingly, at 3 months, and dramatic improvements in hearing function were obtained at all speech frequencies, approaching normal hearing levels without the hearing aid (Figure 5G). The hearing of the ear receiving AAV treatment was comparable to the ear with CI (Figure 5G). Moreover, after 2 months of recovery, this patient was able to hear and respond to sounds with the CI turned off. The little girl could respond correctly to questions such as “What is the color?”, “Where are the eyes of the toy?”, etc. (Movie S3, Supporting Information). Taken together, these data demonstrate that AAV‐*OTOF* could almost completely restore hearing in an auditory neuropathy patient with DFNB9 ≈3 months after injection, with the improved performance being similar to normal hearing.

### 2.4 Hearing was Greatly Improved in an 8‐year‐old Patient After AAV‐*OTOF* Administration to the Bilateral Cochleae

**Figure 6:**
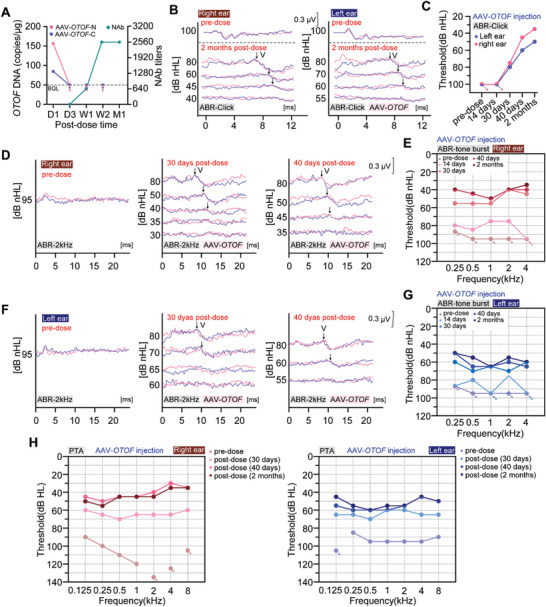
AAV‐*OTOF* rescued hearing in the 8‐year‐old patient with *OTOF* mutations. A) Genomic DNA copies of AAV‐*OTOF*‐N and AAV‐*OTOF*‐C and the NAbs levels from the patient at 1 day (D1), 3 days (D3), 1 week (W1), 2 weeks (W2) and 1 month (M1) after AAV injection, respectively. The arrows indicate that the DNA copies were below the quantification limit (BQL). B) Representative traces recorded for tone‐burst ABR at 2 kHz at 2 months after AAV‐*OTOF* injection. The recording was made twice at each sound intensity. Wave V is indicated by the arrows. C) The click ABR results of the patient. D) Representative traces recorded for tone‐burst ABR at 2 kHz of the right ear from the patient at 30/40 days after AAV‐*OTOF* injection. The recording was made twice at each sound intensity. Wave V is indicated by the arrows. E) The tone‐burst ABR results of right ear from the patient. F,G) Similar results as (D‐E) excepted that the left ear was analyzed. H) The PTA results of the patient before and after AAV‐*OTOF* injection. The arrows in (C, E, G, H) indicate that the patient had no responses to the maximum sound intensity at each frequency.

Besides the 5‐year‐old patient, we included an 8‐year‐old patient, which may reflect a wider therapeutic window for DFNB9. This patient had not undergone cochlear implantation, and AAV‐*OTOF* was injected into both ears. AAV‐*OTOF*‐N and AAV‐*OTOF*‐C DNAs were detected in whole blood on postoperative day 1 (188.59 copies/100 µL for *OTOF*‐N and 84.86 copies/100 µL for *OTOF*‐C) (**Figure** **6**A). From day 3 onward, AAV was no longer detected in the blood, while the amount of NAbs against AAVs in the serum began to increase (Figure 6A), which was similar to the 5‐year old patient. The DPOAE and CM data of the 8‐year‐old patient were also collected, which showed failed DPOAE (Figure S4B, Supporting Information) but successful CM at 2 weeks post‐surgery (Figure S5B, Supporting Information).

After injection, the threshold of click ABR decreased in both in ears, with dramatically decreasing reaching with the threshold restored to 35 dB and 50 dB, respectively (Figure 6B,C). The tone‐burst ABR results showed that, the hearing of the right ear was partially improved at 2 weeks, from unresponsive at the maximum sound intensity to 80, 85, 75, 75, and >95 dB at 0.25, 0.5, 1, 2, 4, and 8 kHz, respectively (Figure 6D,E). After that, the hearing ability of the right ear continued to improve. At 40 days, the hearing thresholds were 40, 45, 50, 40, and 40 dB at 0.25, 0.5, 1, 2, 4, and 8 kHz, respectively, which was similar with those at 2 months (Figure 6E). Similar hearing recovery happened in the left ear (Figure 6F,G). At 2 weeks, the ABR results of the left ear showed an obvious improvement in hearing thresholds to ≈75 dB at 0.5 kHz and 2 kHz (Figure 6G). At 2 months, the full‐frequency hearing improved from no response to sound stimulus before surgery to 50, 55, 65, 55, and 60 dB at 0.25, 0.5, 1, 2, 4, and 8 kHz, respectively (Figure 6G). Thus, these results indicate that the 8‐year‐old patient could hear normal conversational sounds.

We also performed the PTA analyses. PTA thresholds of both ears at most frequencies were ≥90 dB before gene therapy. Only 40 days after AAV‐*OTOF* injection, PTA thresholds were decreased from ≥90 dB to 30–50 dB at human speech frequencies (threshold values: right ear, 45, 50, 45, 45, 40, 30, and 35 dB at 0.125, 0.25, 0.5, 1, 2, 4, and 8 kHz, respectively; left ear, 55, 60, 60, 60, 55, 45, and 50 dB at 0.125, 0.25, 0.5, 1, 2, 4, and 8 kHz, respectively), which was similar with those at 2 months (Figure 6H). We also tested the hearing ability with hearing aids. Before the surgery, the hearing improvement with the hearing aid was only 10–30 dB (Figure S6B, Supporting Information). After gene therapy, the hearing of the injected ears with the hearing aids recovered gradually over time eventually reaching a level similar to that of the normal ear (Figure S6B, Supporting Information). Overall, AAV‐*OTOF* injection partially rescued the hearing of this older patient, indicating a wider treatment window for DFNB9.

### 2.5 Delivery of AAV‐*OTOF* into the Cochleae of the Patients Caused no Changes in Physiological Homeostasis

To evaluate the safety of AAV‐*OTOF* delivery, the patients received post‐operative follow‐up, including physical examination and clinical pathology tests (blood cell counts, blood biochemistry, coagulation function, and urinalysis. No significant physical changes were observed. Several indices were changed, but these were not considered to be clinically significant. Taken together, these results suggest that AAV‐*OTOF* that reaches the blood through the lymphatic circulation does not affect fluid or blood homeostasis.

## 3 Discussion

This study demonstrates the efficacy of AAV‐mediated gene therapy for hereditary DFNB9. Importantly, the treatment is safe with no serious adverse events occurring during treatment. We systematically studied AAV‐*OTOF* gene therapy in mice (Figure 2), cynomolgus macaques (Figure 3), and human patients (Figures 5 and 6). Our study shows that dual AAV‐*OTOF* delivery can effectively express full‐length OTOF protein in mouse IHCs and that it is safe in mice, cynomolgus macaques, and human patients. The safety is demonstrated not only in the auditory system but also in other organs.

DFNB9 patients with OTOF gene mutations have severe to profound prelingual non‐syndromic sensorineural hearing loss and account for 2–8% of all cases of congenital hearing loss.^[^ ^1^ ^]^ About 200 pathogenic or potentially pathogenically recessive mutation sites have been found in *OTOF*‐related deafness.^[^ ^5^ ^]^ Usually, *OTOF* mutations cause congenital or pre‐linguistic severe sensorineural hearing loss, but there have also been reports of atypical hearing phenotypes ranging from mild to moderate hearing loss,^[^ ^6^, ^33^ ^]^ and even temperature‐sensitive hearing loss.^[^ ^34^, ^35^ ^]^ More than 200 pathogenic and probably pathogenic *OTOF* variants have been reported. Most *OTOF* mutation sites are sporadic and unique, which may be one of the reasons why OTOF mutations cause phenotypic diversity. *OTOF* is involved in the release of synaptic vesicles, a key process by which inner hair cells send signals to auditory nerve fibers.

Mutations at different locations may affect the vascular tethering, exocytosis, and reproduction in IHCs, resulting in diverse hearing loss phenotypes. A clinical study by Thorpe et al. explored the association of *OTOF* genotypes with deafness phenotypes and found that biallelic missense variants and indel/missense genotypes cause severe hearing loss.^[^ ^36^ ^]^ The patients included in our study carried compound heterozygous *OTOF* mutations and showed severe hearing loss in both ears, which was consistent with the expression of the above correlation. However, the relationship between phenotype and *OTOF* genotype is poorly understood. Therefore, it is essential to study how *OTOF* mutations impact hearing and to identify the relationship between *OTOF* mutations and heterogeneous clinical phenotypes, which will provide a more detailed research background for clinical studies of *OTOF* gene therapy.

Theoretically, the gene replacement therapy in this study can cover almost all patients with various *OTOF* mutations. However, the treatment window is worth noting. Our research in *OTOF* mutant mice showed that virus injection around P30 can achieve near WT level hearing recovery (Figure 2A–C), but the treatment window in human patients needs to be explored. In this trial, we included two children aged 5 and 8 years, both of whom had remarkable hearing recovery. Although the outcome was slightly better for the 5‐year‐old than for the 8‐year‐old, the delivery of AAV‐*OTOF* in the older patient demonstrated the treatable window, which will benefit clinical trials. In addition, from the comparison of 2‐week and 1‐month data (Figures 5 and 6), it appears to take some time for hearing function to recover. The threshold of click ABR had completely reached the level of normal hearing, but we do not know if the patient's hearing as indicated by tone‐burst ABR and PTA will eventually be fully restored. We should also note that false‐positive responses to the hearing threshold test with a hearing‐aid were evident in the PTA trial in the 8‐year‐old patient (Figure S6B, Supporting Information). As patients age, long‐term follow‐up studies, collecting data from speech tests and comparing the communication abilities with peers of the same age will be necessary.

These two cases were found to have a recessive *OTOF* mutation during screening. Their hearing was almost completely lost, but the DPOAE results showed that her outer hair cells were still functional, suggesting a potentially intact cochlear structure. This is an important indicator for patient screening. However, must be noted that the theoretical DPOAE results of patients with different *OTOF* mutations vary greatly,^[^ ^36^ ^]^ so it is necessary to combine other indexes to determine whether patients are suitable for gene therapy, such as CM as we showed in this study.

Another focus of this study was establishing a safe surgical technique for inner ear delivery in humans. We first established surgical protocols in cynomolgus macaques using endoscope‐assisted round window injection, and hearing was well preserved after injection in six cynomolgus macaques using this technique. In our patients, we found subtle differences in cochlear structure and localization of round window niche compared to cynomolgus macaques, leading to slight variations in injection that may have impacted virus delivery in the patients. Thus, the optimization of the surgical approach requires further study.

The AAV delivery system is derived from natural AAV viruses and comprises two parts, an icosahedral capsid, and the single‐stranded DNA, both of which can have potential toxicity^[^ ^37^, ^38^ ^]^ and thus limit the application of AAV‐based gene therapy. Although the short‐term safety in mice, cynomolgus macaques, and our patient has been demonstrated, the long‐term safety remains to be studied. Importantly, elevated AAV DNA was detected in blood one day after local injection into the cochlea in the patients and cynomolgus macaques (Figures 5A and 6A; Table S2, Supporting Information), indicating that we should consider side effects on immunity and on other organs. Indeed, we observed AAV accumulation in the brain and liver after inner ear delivery (Table S1, Supporting Information), although no toxicity was observed. These findings indicate that multiple organs should also be monitored besides the auditory system in long‐term patient follow‐up.

Our study suggests that AAV gene therapy has the potential to restore the hearing function of deafness patients. It is unknown how long the therapeutic effect will last, although other studies show that AAV‐mediated transgene expression can be maintained for several years in muscle and other organs.^[^ ^39^ ^]^ However, loss of expression has also been reported. AAV re‐injection may be a way to maintain the effect of gene therapies, but the immune response – especially the neutralizing antibodies for AAV vectors – may hinder the effect of AAV re‐dosing. Eliciting the pre‐existing neutralizing antibodies with IgG‐cleaving endopeptidase ^[^ ^40^ ^]^ before AAV re‐injection or using another AAV serotype may support AAV re‐dosing and maintain the therapeutic effect. However, there is no lymphatic drainage of the cochlea, and the circulation and the cochlear microenvironment is separated by the blood‐labyrinth barrier, suggesting that the inner ear maybe an immune‐privileged organ.^[^ ^41^ ^]^ Thus, this leaves a possibility that AAV re‐injection in the inner ear may be not affected by the immune response. More experiments need to be performed to elucidate the immune response in the inner ear.

Although looking at only two cases, this study is the first to demonstrate the feasibility of AAV‐mediated gene therapy for hearing loss. More cases will be recruited to explore and optimize the treatment window, dose, volume, and surgical method in order to achieve better treatment effects. Additionally, long‐term follow‐up will determine the safety of the AAV vector itself and the delivered transgene. The implementation of findings from these studies will have an impact on the entire field of gene therapy for deafness and will provide references for gene therapies targeting other organs.

## 4 Experimental Section

### Animals

Animals of both sexes were used in this study. Mice were housed in an SFP‐class animal facility with a constant temperature and humidity and with a 12 h light‐dark cycle. The genotyping primers for OTOF^Q939*/Q939*^ mice were: Forward, 5′‐TAT CTA CCT CTG CCT AAA GCT TCA AC‐3′ and Reverse, 5′‐GCT CTG GTT GAT GAA GAA GAC AC‐3′. All mouse experiments were approved by the Institutional Animal Care and Use Committee of Southeast University, China, and all experimental procedures in cynomolgus macaques were performed and approved by the Animal Care and Use Committee at JOINN Laboratories Co., Ltd. (ethical approval number: S‐ACU23‐2563).

### AAV‐*OTOF* Design

The therapeutic AAV vector contained the mouse hair cell‐specific promoter and the human *OTOF* gene, as well as the SD/AS and AK sequences for mediating recombination. The full‐length *OTOF* gene was constructed as two vectors splitting between exons. *OTOF‐N* and *OTOF‐C* were packaged in an AAV capsid with high transduction rate of IHCs in adult mice. The details are shown in Figure 1A.

### Inner Ear Injection in Mice

Adult mice were anesthetized by intraperitoneal injection of tribromoethanol (500 mg kg^−1^). The hair around the left ear and neck area of the mouse was shaved, and a small incision was made ≈0.5 cm behind the ear. Fat and muscle were dissected under a microscope until the posterior semicircular canal was exposed. Then a small hole was poked in the center of the posterior semicircular canal using the beveled tip of a needle. AAV‐*OTOF*‐N and AAV‐*OTOF*‐C were mixed 1:1 by vg for injection. A total of 1–2 µL virus was slowly injected into each cochlea through the hole via a glass electrode, and the hole was plugged with a small piece of muscle. The exposed wound was sutured, and the mouse was placed on a 38 °C heating pad until awake.

### ABR Measurement in Mice

Adult mice were anesthetized by intraperitoneal injection of tribromoethanol (500 mg kg^−1^). The recording electrode, negative electrode, and reference electrode were inserted subcutaneously at the vertex, behind the test ear, and behind the contralateral ear, respectively. During the ABR tests, tones of different frequencies (Click, 4, 8, 12, 16, and 32 kHz) and different sound intensities (20–90 dB) were generated and delivered using a TDT RZ6 auditory physiology workstation. The lowest sound stimulus that elicited ABRs was considered to be the threshold.

### Immunofluorescence Staining

Samples were immunostained as previously described.^[^ ^30^ ^]^ Samples were fixed in 4% PFA, decalcified in 0.5 M EDTA, permeated in 0.5% Triton‐X100, and blocked in 10% donkey serum. The antibodies were against OTOF‐N (Abcam, 1:200 dilution, ab53233), OTOF‐C (Invitrogen, 1:200 dilution, PA552935), and Phalloidin (Invitrogen, 1:1000 dilution, A22287). Confocal images were captured on a Zeiss LSM900.

### Inner Ear Injection in Cynomolgus Macaques

Animals were anesthetized with propofol (5 mg kg^−1^ min^−1^) intravenously and maintained at 0.1‐0.5 mg kg^−1^ min^−1^. After intubation, isoflurane was used to maintain anesthesia. The round window membrane was exposed surgically and injected under an ear endoscope. AAV‐*OTOF*‐N and AAV‐*OTOF*‐C were mixed 1:1 by volume for injection. The solution was delivered into the inner ear using a microinjection system (WPI, nano 3000). Analgesics and antibiotics were administered after surgery.

### ABR Measurement in Cynomolgus Macaques

Cynomolgus macaques were anesthetized with propofol (5 mg kg^−1^ min^−1^) intravenously and maintained at 0.1–0.5 mg kg^−1^ min^−1^. Electrodes were subcutaneously inserted at the vertex and behind the pinna on both sides. For the ABR tests, a Neuro‐Audio auditory physiology workstation (Neurosoft) was used to generate and deliver tones of different frequencies (Click, 0.5, 1, 2, 4, 8, 12, and 16 kHz) and different sound intensities (20–90 dB). The lowest sound stimulus that elicited ABRs was taken as the threshold.

### Hematoxylin‐Eosin Staining

Samples were analyzed at JOINN Laboratories Co., Ltd. The samples were fixed in 4% PFA and dehydrated in graded ethanol (30%, 50%, 70%, 80%, 90%, 95%, 100%). The samples were then cleared in xylene, embedded in paraffin, sectioned, and stained with hematoxylin and eosin. Images were captured on a Leica fluorescence microscope (Leica, DMI8).

### DNA Extraction and qPCR Analyses

≈20 mg tissue or 0.5–1 mL whole blood was homogenized in cell lysis buffer (MagMAX DNA Cell and Tissue Extraction Buffer) followed by addition of RNase A and Proteinase K. DNA was extracted using the MagMAX DNA Multi‐Sample Ultra 2.0 Kit (Thermo Fisher). Tissue DNA was quantified by qPCR. Standard curves were generated using dual vector plasmids. The qPCR was performed on a QuantStudio Design, and copy numbers were calculated from the standard curves. Primers and probes for *OTOF* were as follow:

For the *N*‐terminus of *OTOF*: (F) 5′‐GGA GCT GGT AAG TAT CAA GGT‐3′; (R) 5′‐GAA ATC GGC AAA ATC CCA GA‐3′; Probe: FAM‐ACT GGG CTT GTC GAG ACA GAG AAG ACT‐BHQ1, and for the C‐terminus of *OTOF*: (F) 5′‐TAC AAT TCA CGC GTG CTA GC‐3′; (R) 5′‐ TCC TGT GGA GAG AAA GGC AA‐3′; Probe: FAM‐GCA CCT ATT GGT CTT ACT GAC‐MGB.

### AAV Administration in the Patients

In 2023, a single round window membrane injection of AAV‐*OTOF*‐N and AAV‐*OTOF*‐C was performed in the ears at a dose of 5.6 × 10^11^ vg or 1.12 × 10^12^ vg for both viruses at Shandong Second Provincial General Hospital (Jinan, China). The patients were discharged on day 7 after surgery without complications.

### ABR Measurement in the Patients

The ABR test was performed when the patients were asleep. Sounds were delivered via headphones, and signals were recorded via electrodes in contact with the skin to determine the thresholds, including click ABR (mainly reflecting 2–4 kHz) and tone‐burst ABR (0.125, 0.25, 0.5, 1, 2, 4, and 8 kHz). For each sound intensity, two records were obtained to determine the final response.

### PTA Measurement in the Patients

PTA was used to evaluate the hearing thresholds at each frequency and is the lowest sound pressure level at which the patient can detect at least 50% of the sound signals. When the patient responded correctly, the sound intensity was lowered by 10 dB until no response, then increased in 5 dB steps until a response reappeared, then lowered again by 10 dB again, and the process was repeated. The PTA threshold is defined as the minimum intensity of correct responses to at least half of the acoustic signals in the rising process, that is, a correct response is one given at least two times for three acoustic stimuli of a certain intensity.

### Statistical Analysis

All the images were processed linearly using ImageJ software (Fiji, Inc.). The data were analyzed using GraphPad Prism 8 software and presented as the mean with standard error of mean. The two‐tailed unpaired Student's *t*‐test or one‐way ANOVA was used to determine the statistical significance of differences. *p* <0.05 was considered statistically significant.

## Conflict of Interest

Z.Z., W.D., L.J., C.Y., J.L., L.W., and C.T. are paid employees of Otovia Therapeutics Inc. S.S. and H.S. are paid employees of Otovia Therapeutics Inc. and Fosun Health Capital. Other authors declare no conflict of interest.

## Author Contributions

J.Q., F.T., L.Z., L.L., S.Z., Y.Z., Y.L., and X.Q. contributed equally to this work. R.C., H.L., X.G., L.X., and S.Y. conceived and designed the experiments. J.Q., F.T., L.Z., S.Z., Y.Z., and Y.L. performed most of the experiments except that L.L. and X.Q. performed the AAV injection in the cynomolgus macaques and L.X. performed the AAV injection in the patients. W.D. helped with the clinical studies. Y.Z., Z.Z., X.Y., L.J., C.Y., J.L., T.C., and L.W., helped with the experiments in mice and cynomolgus macaques. C.T. helped with the GMP‐AAV production. J.Q. and L.Z. analyzed and presented the data. S.S. and H.S. helped with the data interpretation and presentation. R.C., J.Q., F.T., and L.Z. discussed the data analysis, interpretation, and presentation and wrote the manuscript with contributions from all authors.

## Supporting information

Supporting Information

Supplemental Movie1

Supplemental Movie2

Supplemental Movie3

## Data Availability

The data that support the findings of this study are available from the corresponding author upon reasonable request.
