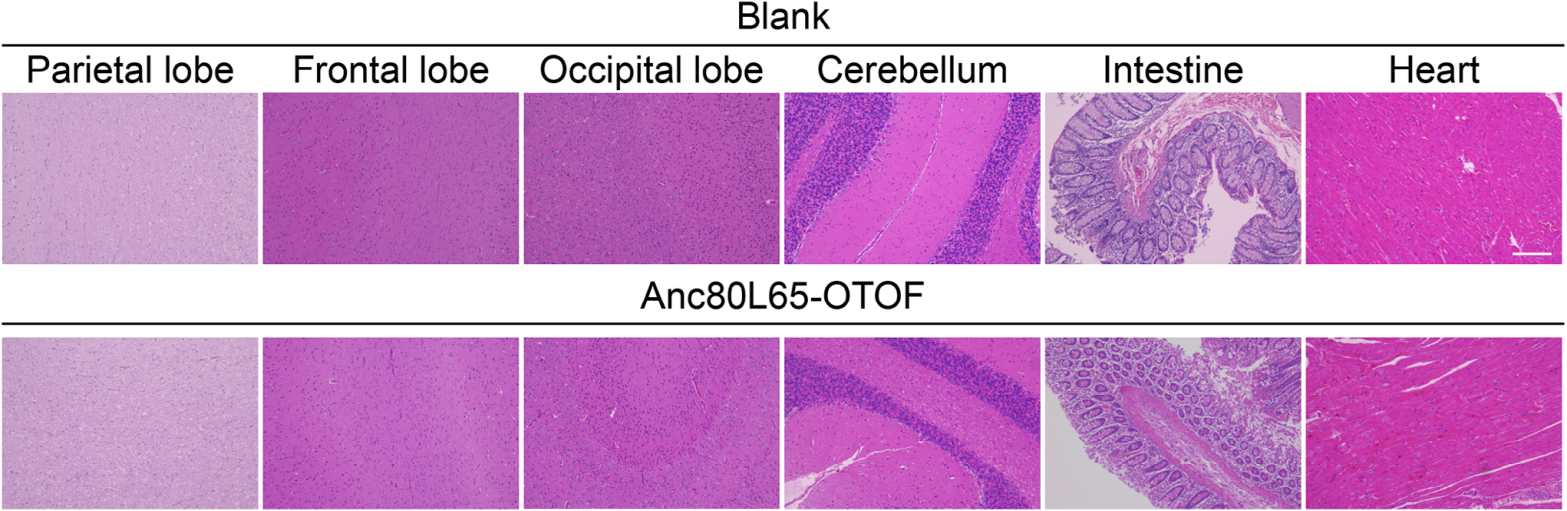# Correction to “AAV‐Mediated Gene Therapy Restores Hearing in Patients with DFNB9 Deafness”

**DOI:** 10.1002/advs.202504047

**Published:** 2025-04-30

Jieyu Qi, Fangzhi Tan, Liyan Zhang, Ling Lu, Shanzhong Zhang, Yabo Zhai, Yicheng Lu, Xiaoyun Qian, WenXiu Dong, Yinyi Zhou, Ziyu Zhang, Xuehan Yang, Lulu Jiang, Chaorong Yu, Jiancheng Liu, Tian Chen, Lianqiu Wu, Chang Tan, Sijie Sun, Huaien Song, Yilai Shu, Lei Xu, Xia Gao, Huawei Li, Renjie Chai. *Adv Sci (Weinh). 2024 Mar;11(11):e2306788*.

Doi: 10.1002/advs.202306788

Description of the error:

In the original published paper, we found that the HE image of the parietal lobe in the control group in Figure S3 (Supporting Information) was improperly used. The corrected figure is shown below. This correction does not affect the overall findings and conclusions of this paper.

We apologize for this error.

Corrected Figure S3 (Supporting Information):