# Supplementary material for: AAV‐Mediated Gene Therapy Restores Hearing in Patients with DFNB9 Deafness
Source: Adv Sci (Weinh). 2024 Jan 8;11(11):2306788. doi: 10.1002/advs.202306788 (PMC10953563; doi:10.1002/advs.202306788)
Supplement: Supplementary file 1 — Supporting Information [file ADVS-11-2306788-s002.pdf]

ADVANCED SCIENCE
Open Access

Supporting Information

for *Adv. Sci.*, DOI 10.1002/advs.202306788

AAV-Mediated Gene Therapy Restores Hearing in Patients with DFNB9 Deafness

Jieyu Qi, Fangzhi Tan, Liyan Zhang, Ling Lu, Shanzhong Zhang, Yabo Zhai, Yicheng Lu, Xiaoyun Qian, WenXiu Dong, Yinyi Zhou, Ziyu Zhang, Xuehan Yang, Lulu Jiang, Chaorong Yu, Jiancheng Liu, Tian Chen, Lianqiu Wu, Chang Tan, Sijie Sun, Huaien Song, Yilai Shu, Lei Xu*, Xia Gao*, Huawei Li* and Renjie Chai**

Supplementary materials for

AAV-mediated gene therapy restores hearing in patients with DFNB9 deafness

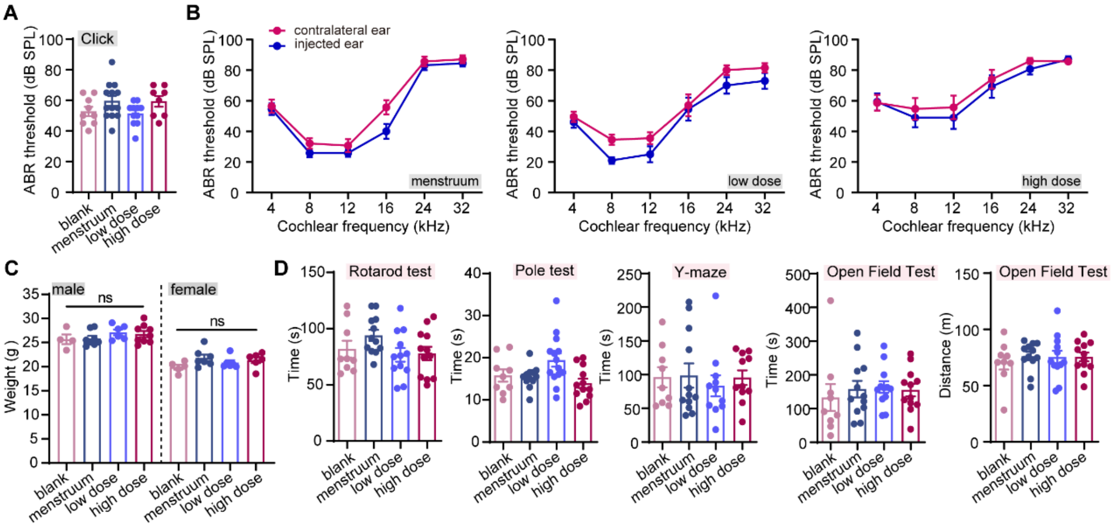

Figure S1. AAV-*OTOF* had no adverse effect in adult WT mice.

(**A-B**) Click and tone-burst ABRs of adult WT mice and WT mice injected with menstruum, low dose AAV-*OTOF*, and high dose AAV-*OTOF* at 3 months after injection. (**C**) The body weight of adult WT mice and WT mice injected with menstruum and virus at 3 months after injection. (**D**) Behavior tests from WT mice and WT mice injected with virus at 3 months after injection. Error bars indicate the standard error of the mean. The *p*-value was calculated by Student's *t*-test or one-way ANOVA. n.s. means no significant difference.

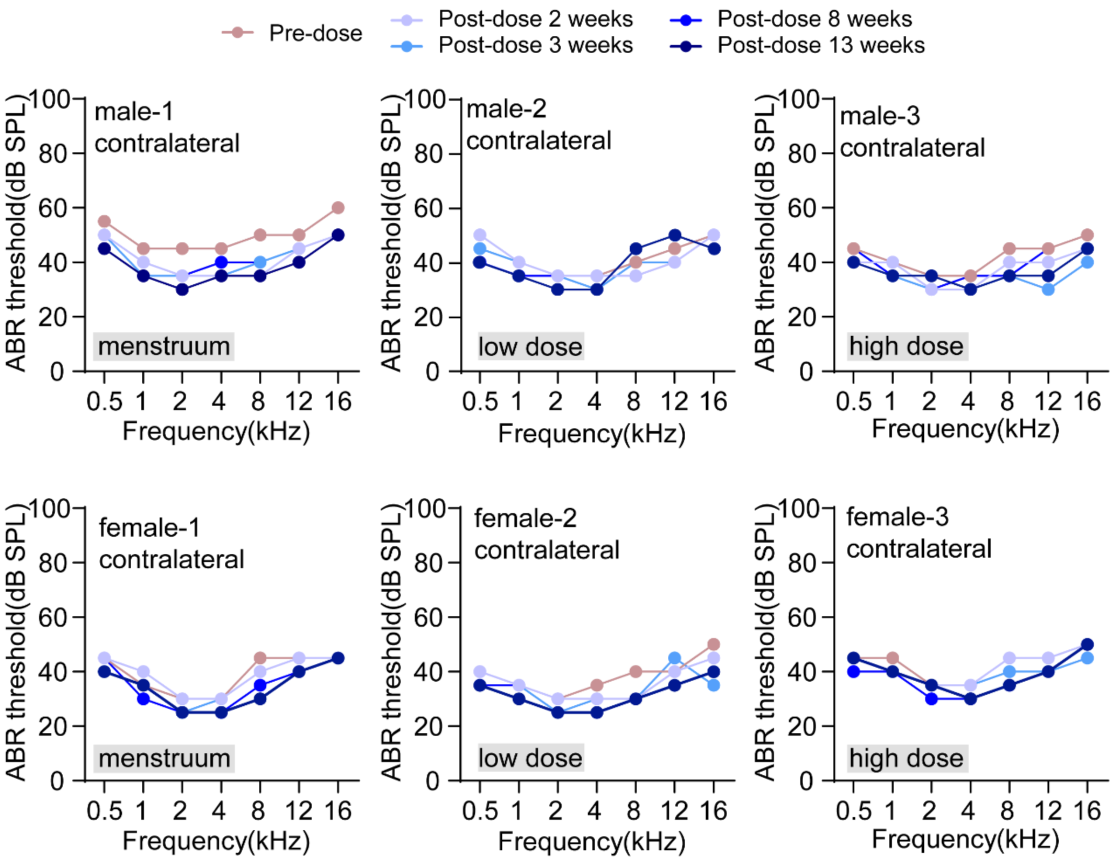

Figure S2. AAV-*OTOF* was safe for the auditory system in the contralateral ear of cynomolgus macaques. The ABR results for the contralateral ear in cynomolgus macaques injected with menstruum, low dose AAV-*OTOF*, or high dose AAV-*OTOF* at 2 weeks, 3 weeks, 8 weeks, and 13 weeks after injection, respectively.

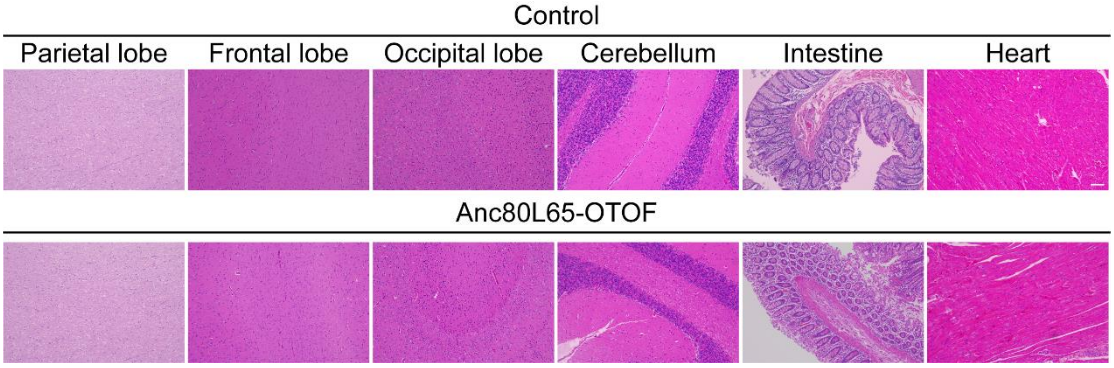

Figure S3. AAV-*OTOF* had no impact on the tissues of cynomolgus macaques. The HE staining of tissues from control or AAV-*OTOF* injected cynomolgus macaques. Scale bar: 100 μm.

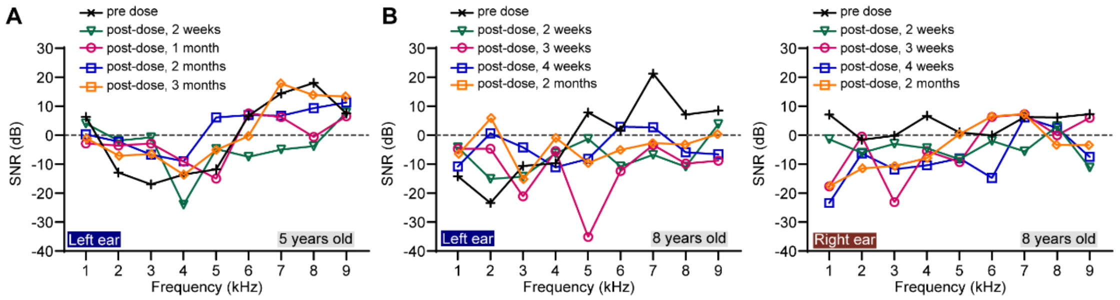

Figure S4. DPOAE results of the two patients before and after AAV-*OTOF* injection.

DPOAE results of AAV-*OTOF* injected ears from the 5-year-old patient **(A)** and 8-year-old patient **(B)**.

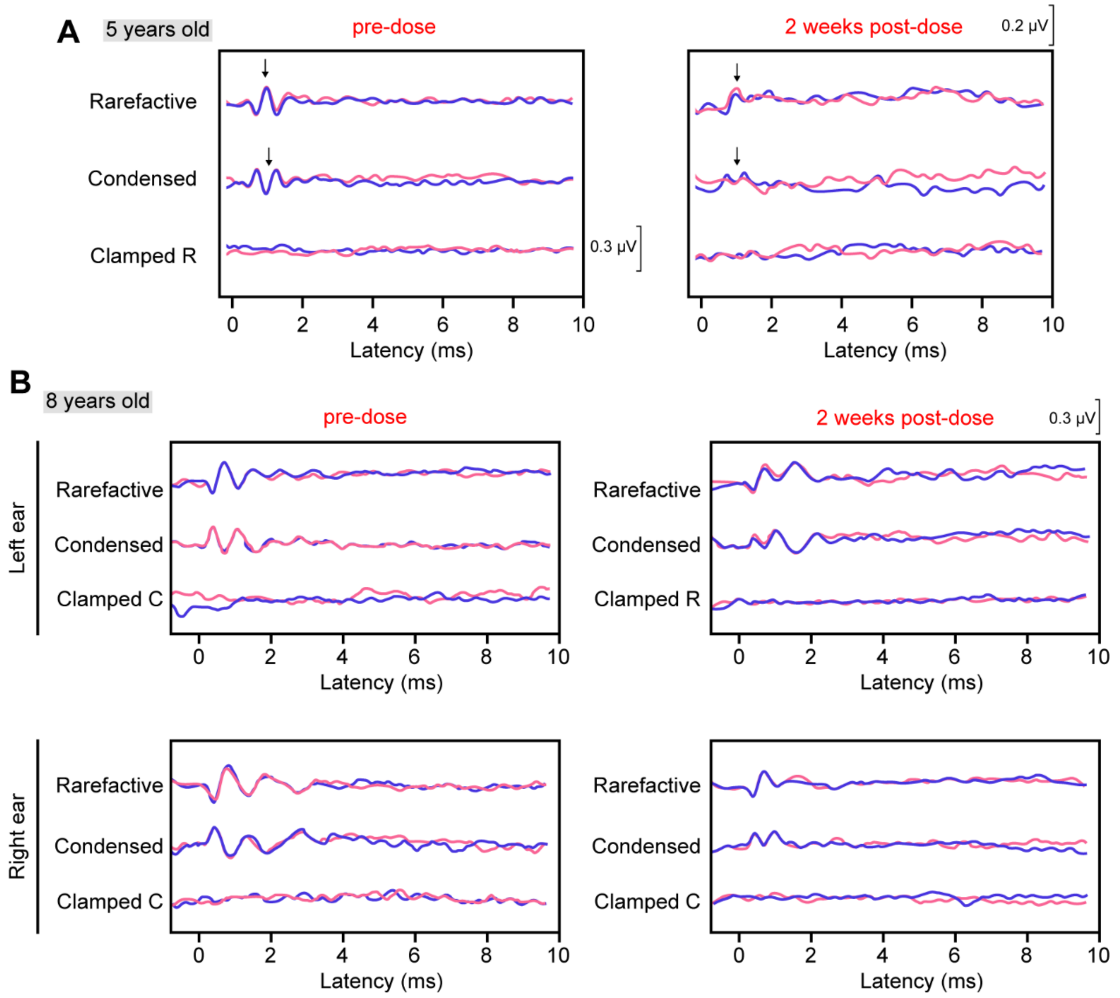

Figure S5. CM results of the two patients after AAV-*OTOF* injection at 2 weeks.

Click CM results of AAV-*OTOF* injected ears from the 5-year-old patient **(A)** and 8-year-old patient **(B)**. The recording was made twice at each sound intensity.

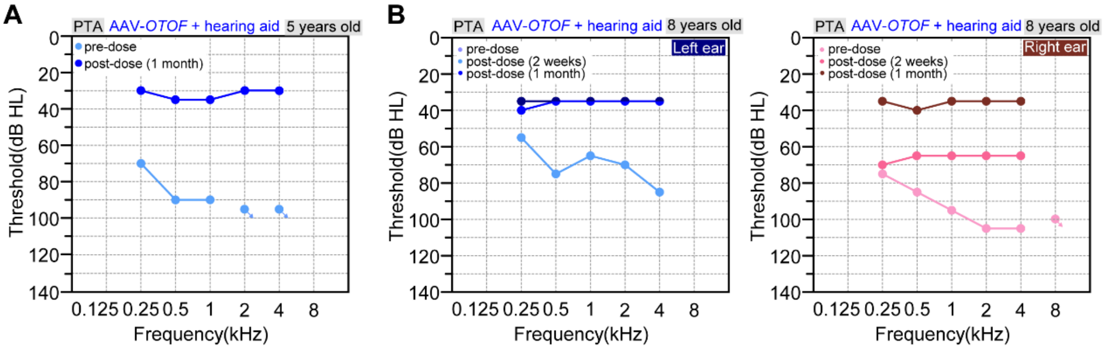

Figure S6. PTA results of the patients with a hearing-aid after AAV-*OTOF* injection. The PTA thresholds of the injected ears with hearing aid from the 5-year-old patient **(A)** and 8-year-old patient **(B)**. The arrows indicate that the patient had no responses to the maximum sound intensity at each frequency.

Movie S1. AAV injection in NHPs.

Movie S2. AAV injection in the patient.

Movie S3. Talk to the patient 2 months after AAV injection.
Here is the description of the movie:

Time: 00:10-01:15
Closed the CI.

Time: 00:23-00:27
The mother asked her daughter (patient) to say the color of the purple rabbit doll, and the daughter answered purple.

Time: 00:40-01:54
The mother asked her daughter (patient) to point out the rabbit's ears, eyes and nose, and the daughter correctly pointed out the position of the rabbit's ears, eyes and nose and responded accordingly.

Table S1. Quantification of genomic DNA of AAV-*OTOF* in the blood and tissues of AAV-*OTOF*-injected mice.

Time	Sex	Group	Animal IDs/Tissues	The number of *OTOF*-N copies/μg										
				Whole blood	Left brain	Right brain	Left cochlea	Right cochlea	Brainstem	Cervical lymph nodes	Heart	Kidney	Liver	Lung
D49	male	low dose	1-1-1	BQL	BQL	BQL	6.07E+03	BQL	BQL	BQL	BQL	BQL	6.13E+02	BQL
			1-1-2	BQL	BQL	BQL	2.53E+04	BQL	BQL	3.24E+02	BQL	BQL	2.63E+03	BQL
		high dose	1-2-1	BQL	1.70E+02	BQL	8.99E+03	BQL	4.20E+02	BQL	BQL	BQL	1.90E+04	BQL
			1-2-1	BQL	BQL	3.35E+02	1.87E+04	6.45E+02	9.86E+02	3.79E+03	9.96E+01	8.33E+01	7.64E+04	5.27E+01
	female	low dose	2-1-1	BQL	BQL	BQL	6.12E+02	BQL	1.20E+02	3.76E+01	BQL	BQL	1.51E+03	BQL
			2-1-2	BQL	BQL	BQL	3.77E+01	9.55E+01	4.77E+02	4.00E+02	BQL	BQL	9.53E+03	BQL
		high dose	2-2-1	BQL	1.00E+03	1.56E+03	7.09E+04	1.79E+03	9.91E+02	6.00E+01	5.47E+01	3.33E+01	2.66E+04	BQL
			2-2-2	BQL	1.48E+02	BQL	2.28E+04	6.14E+02	4.93E+02	1.28E+04	BQL	BQL	1.88E+04	BQL
D92	male	low dose	3-1-1	BQL	BQL	BQL	1.57E+04	1.03E+01	BQL	7.16E+01	BQL	BQL	3.06E+03	BQL
			3-1-2	BQL	8.30E+02	BQL	8.95E+03	2.74E+01	BQL	5.80E+01	BQL	BQL	6.16E+03	BQL
		high dose	3-2-1	BQL	BQL	8.22E+01	9.28E+04	1.50E+03	1.27E+03	2.45E+02	4.18E+02	BQL	6.98E+04	4.97E+01
			3-2-2	BQL	3.59E+03	1.11E+02	4.68E+03	4.59E+03	1.88E+03	6.34E+02	2.93E+01	2.36E+01	5.74E+04	BQL
	female	low dose	4-1-1	BQL	BQL	BQL	1.86E+03	BQL	3.19E+02	6.06E+02	BQL	BQL	1.51E+03	BQL
			4-1-2	BQL	BQL	4.12E+02	1.10E+04	2.73E+02	1.89E+03	9.30E+02	BQL	BQL	2.47E+03	BQL
		high dose	4-2-1	BQL	2.96E+03	3.09E+03	1.67E+04	8.79E+02	2.19E+04	3.79E+04	2.41E+02	9.88E+01	8.73E+04	1.28E+02
			4-2-2	BQL	1.33E+02	1.26E+02	6.19E+03	7.82E+02	6.88E+02	1.75E+03	BQL	BQL	1.80E+04	3.31E+01

Time	Sex	Group	Animal IDs/Tissues	The number of *OTOF*-C copies/μg										
				Whole blood	Left brain	Right brain	Left cochlea	Right cochlea	Brainstem	Cervical lymph nodes	Heart	Kidney	Liver	Lung
D49	male	low dose	1-1-1	BQL	BQL	BQL	8.01E+03	BQL	BQL	BQL	BQL	BQL	5.97E+02	BQL

Time	Sex	Group	Animal IDs/Tissues	**The number of *OTOF*-C copies/μg**										
				Whole blood	Left brain	Right brain	Left cochlea	Right cochlea	Brainstem	Cervical lymph nodes	Heart	Kidney	Liver	Lung
			1-1-2	BQL	1.09E+02	BQL	2.60E+04	BQL	BQL	2.24E+02	BQL	BQL	2.75E+03	BQL
		high dose	1-2-1	BQL	2.91E+02	BQL	1.30E+04	1.96E+02	6.56E+02	BQL	BQL	3.89E+01	2.07E+04	BQL
			1-2-1	6.75E+02	BQL	4.67E+02	2.09E+04	8.63E+02	1.29E+03	2.61E+03	2.28E+02	1.44E+02	6.95E+04	8.04E+01
	female	low dose	2-1-1	BQL	BQL	BQL	6.93E+02	BQL	1.90E+02	3.81E+01	BQL	BQL	1.90E+03	BQL
			2-1-2	BQL	BQL	BQL	BQL	1.12E+02	6.28E+02	2.76E+02	BQL	BQL	9.37E+03	BQL
		high dose	2-2-1	BQL	1.40E+03	1.87E+03	8.87E+04	2.74E+03	1.78E+03	4.98E+01	9.29E+01	8.35E+01	4.23E+04	3.32E+01
			2-2-2	BQL	2.87E+02	BQL	2.94E+04	8.49E+02	8.44E+02	1.05E+04	BQL	BQL	2.78E+04	BQL
D92	male	low dose	3-1-1	BQL	BQL	BQL	1.32E+04	9.45E+00	BQL	3.67E+01	BQL	BQL	1.84E+03	BQL
			3-1-2	BQL	6.81E+02	BQL	5.72E+03	3.75E+01	BQL	7.20E+01	BQL	BQL	3.69E+03	BQL
		high dose	3-2-1	BQL	BQL	6.92E+01	8.05E+04	1.61E+03	1.26E+03	1.40E+02	3.61E+02	5.09E+01	6.52E+04	4.30E+01
			3-2-2	BQL	3.82E+03	1.22E+02	3.20E+03	6.95E+03	2.21E+03	3.20E+02	3.90E+01	2.25E+01	4.38E+04	4.45E+01
	female	low dose	4-1-1	BQL	BQL	BQL	1.09E+03	BQL	3.38E+02	3.87E+02	BQL	BQL	1.50E+03	BQL
			4-1-2	BQL	BQL	4.57E+02	9.38E+03	2.16E+02	2.03E+03	1.06E+03	BQL	BQL	1.83E+03	BQL
		high dose	4-2-1	BQL	2.70E+03	2.77E+03	1.55E+04	7.79E+02	2.21E+04	2.87E+04	3.08E+02	9.18E+01	7.40E+04	1.04E+02
			4-2-2	BQL	1.52E+02	1.08E+02	5.71E+03	7.67E+02	6.56E+02	7.64E+02	BQL	BQL	1.96E+04	3.87E+01

BQL: below the quantification limit (50 copies/μg)

Table S2. Quantification of the genomic DNA of AAV-*OTOF* in the blood of cynomolgus macaques.

***OTOF*-N copies/μg**										
Group	Gender	No./time	Blood							
			pre-dose	D1	D4	D8	D15	D29	D57	D92
menstruum	male	2345721	BQL	BQL	BQL	BQL	BQL	BQL	BQL	BQL
	female	2345722	BQL	BQL	BQL	BQL	BQL	BQL	BQL	BQL
low dose	male	2345723	BQL	6.87E+01	BQL	BQL	BQL	BQL	BQL	BQL
	female	2345724	BQL	2.36E+02	BQL	BQL	BQL	BQL	BQL	BQL

high dose	male	2345725	BQL	5.84E+05	3.35E+03	BQL	BQL	BQL	BQL	BQL
	female	2345726	BQL	9.50E+02	BQL	BQL	BQL	BQL	BQL	BQL

***OTOF*-C copies/μg**										
Group	Gender	No./time	Blood							
			pre-dose	D1	D4	D8	D15	D29	D57	D92
menstruum	male	2345721	BQL	BQL	BQL	BQL	BQL	BQL	BQL	BQL
	female	2345722	BQL	BQL	BQL	BQL	BQL	BQL	BQL	BQL
low dose	male	2345723	BQL	7.57E+01	BQL	BQL	BQL	BQL	BQL	BQL
	female	2345724	BQL	2.99E+02	BQL	BQL	BQL	BQL	BQL	BQL
high dose	male	2345725	BQL	4.40E+05	2.93E+03	BQL	BQL	BQL	BQL	BQL
	female	2345726	BQL	1.05E+03	BQL	BQL	BQL	BQL	BQL	BQL

BQL: below the quantification limit (50 copies/μg).
